# Recommendations for older adults’ physical activity and sedentary behaviour during hospitalisation for an acute medical illness: an international Delphi study

**DOI:** 10.1186/s12966-020-00970-3

**Published:** 2020-05-25

**Authors:** Claire E. Baldwin, Anna C. Phillips, Sarah M. Edney, Lucy K. Lewis

**Affiliations:** 1 Caring Futures Institute, College of Nursing and Health Sciences, Flinders University, Flinders Drive, Bedford Park, Adelaide, South Australia 5042 Australia; 2 Allied Health and Human Performance, University of South Australia, Adelaide, Australia; 3 Sport, Health, Activity, Performance and Exercise (SHAPE) Research Centre, Flinders University, Adelaide, Australia

**Keywords:** Physical activity, Sedentary behaviour, Ageing, Hospitalisation, Clinical guideline, Physiotherapy, Acute illness, Older adult, Delphi

## Abstract

**Background:**

Immobility is major contributor to poor outcomes for older people during hospitalisation with an acute medical illness. Yet currently there is no specific mobility guidance for this population, to facilitate sustainable changes in practice. This study aimed to generate draft physical activity (PA) and sedentary behaviour (SB) recommendations for older adults’ during hospitalisation for an acute medical illness.

**Methods:**

A 4-Round online Delphi consensus survey was conducted. International researchers, medical/nursing/physiotherapy clinicians, academics from national PA/SB guideline development teams, and patients were invited to participate. Round 1 sought responses to open-ended questions. In Rounds 2–3, participants rated the importance of items using a Likert scale (1–9); consensus was defined a priori as: ≥70% of respondents rating an item as “critical” (score ≥ 7) and ≤ 15% of respondents rating an item as “not important” (score ≤ 3). Round 4 invited participants to comment on draft statements derived from responses to Rounds 1–3; Round 4 responses subsequently informed final drafting of recommendations.

**Results:**

Forty-nine people from nine countries were invited to each Round; response rates were 94, 90, 85 and 81% from Rounds 1–4 respectively. 43 concepts (items) from Rounds 2 and 3 were incorporated into 29 statements under themes of PA, SB, people and organisational factors in Round 4. Examples of the final draft recommendations (being the revised version of statements with highest participant endorsement under each theme) were: “*some PA is better than none”,* “*older adults should aim to minimise long periods of uninterrupted SB during waking hours while hospitalised”, “when encouraging PA and minimising SB, people should be culturally responsive and mindful of older adults’ physical and mental capabilities”* and *“opportunities for PA and minimising SB should be incorporated into the daily care of older adults with a focus on function, independence and activities of daily living”.*

**Conclusions:**

These world-first consensus-based statements from expert and stakeholder consultation provide the starting point for recommendations to address PA and SB for older adults hospitalised with an acute medical illness. Further consultation and evidence review will enable validation of these draft recommendations with examples to improve their specificity and translation to clinical practice.

## Background

Inactivity impacts the morbidity and mortality of conditions such as heart disease, stroke and diabetes [1] that are common in people admitted to hospital, either as the presenting complaint or as a concurrent condition. A high proportion of older adults’ experience both the disease burden of low physical activity (PA, being any bodily movement produced by skeletal muscles that requires energy expenditure) [2] and hospital admissions. The Australian Burden of Disease Study reported that people aged 65 to 74 years represented 22% of the overall physical inactivity burden, people aged 75 to 84 years contributed to 25% and the 85+ age group contributed to 18% [1]. In 2017–18, 42% of hospitalisations in Australia were people aged 65 years and over, a rate which is increasing beyond population growth for this age group [3]. Acute hospital cost savings have been associated with people and particularly older adults’ meeting the PA guideline [2] of engaging in at least 150 min of moderate-intensity PA throughout the week [4]. Similarly, hospital bed days may be reduced for older adults with greater daily step counts [5]. Yet 75% of community dwelling Australians aged 65 years and over do not meet the PA guideline of sufficient PA by engaging in at least 30 min of PA on ≥5 days per week [6].

The problems associated with low levels of PA are not unique to Australia, they are of global relevance as indicated by economic [7] and observational data [6, 8]. Furthermore, the multi-disciplinary #endpjparalysis movement has been widely promoted and highlights both awareness and action to get people in hospital up, dressed, and moving, with the goal of reducing immobility and protecting dignity [9]. Sedentary behaviour (SB, defined as the waking time spent sitting or lying down, or, according to an energy expenditure threshold of ≤1.5 METs) [10] is prevalent in acute hospitals; with adults spending 87–100% of their time in hospital in seated or lying postures [11, 12]. When hospitalised with an acute medical illness, older adults are at high risk of functional decline, newly acquired disabilities and poor outcomes that persist post-hospitalisation such as continued decline, institutionalisation and death [13–15]. Research is only beginning to investigate activity dosage in older medical patients with reports of: a reduced risk of 30-day hospital readmission above a threshold of 275 steps per day and further risk reduction for every 100 step increase in mean daily steps [16]; an increased risk of hospital associated functional decline if taking ≤900 steps per day [17, 18] and the suggestion that walking at least twice a day for 20 min is associated with less functional decline in people of variable physical capabilities [19]. The problems of low mobility and SB in hospitals are complex because there are system issues, in addition to challenges relating to people, culture, the environment and operational processes [20]. At the individual level, complexities can relate to the heterogeneity of acute medical conditions (clinical stability and the safety of exercise in different populations) and levels of independence seen in older adults. There is some evidence for mobilisation and exercise programs (including within models of care such as ‘Acute Care for Elders’) [21–24] but there remain few interventions [25–27] to address low mobility and high levels of SB for people in hospitals [28].

Support for clinical practice in the form of protocols or guidelines on PA and SB for the acute hospital setting is also lacking. While surgical patient groups are covered by acute post-operative mobility protocols or ‘Enhanced Recovery After Surgery’ (ERAS) pathways [29], these do not apply to older adults who are admitted to hospital with an acute medical illness. Whether components of existing guidelines [30] remain applicable when older adults are acutely hospitalised with a medical illness is not known. The development of guidelines or recommendations needs to be rigorous and transparent. The Grading of Recommendations Assessment, Development and Evaluation (GRADE) approach is a method for rating the strength and quality of evidence when making recommendations [31]. Alternatively, the Delphi methodology can be used to shape the field of research, accelerate progress and prevent pitfalls in future intervention studies [32] for research areas where the body of empirical data on interventions are unavailable or inadequate. The Delphi methodology is well-established and allows for the collection of expert and stakeholder opinion and consensus agreement on a topic, through prospective surveys [33]. Given the importance of activity for older people in hospital, and the lack of existing guidance, this study aimed to gain consensus opinion from a range of international stakeholders on older adults’ PA and SB during hospitalisation for an acute medical illness, with the goal of drafting recommendations and targets.

## Methods

This study used an online Delphi survey methodology. The research is compliant with the ‘Recommendations for the Conducting and REporting of DElphi Studies’ (CREDES) [34]. The project was prospectively registered (COMET Initiative: www.comet-initiative.org/studies/details/1338?result=true) and approved by the Flinders University, Social and Behavioural Research Ethics Committee (SBREC) (project 8254) including procedures for informed consent.

### Participants and panel recruitment

Participants were sought from a range of stakeholder groups [35], being researchers, clinicians, policy makers and patients (recruitment strategy in Additional file 1, Table S1). Researchers were corresponding authors identified from publications describing PA and/or SB in hospitalised older adults with an acute medical illness [11, 36–38], or, were purposively selected on recommendation from other participants and researcher networks for their expertise in PA/SB and management in the acute setting of: chronic obstructive pulmonary disease, chronic heart failure, stroke or cancer. Clinicians were nominated by professional associations for physiotherapy, medicine, and nursing from Australia, Canada, the United Kingdom and the United States. The stakeholder group of policy makers was formed from academics who were associated with national Australian, Canadian, United Kingdom and United States PA and/or SB Guideline development teams [39–42], and nominated representatives from international societies that advocate for improving PA and reducing SB. Patient stakeholders were recruited from Australia via social media and a health consumer newsletter, being eligible if they were aged ≥65 years and had been hospitalised with a medical illness (not elective surgery) for at least one night in the last two years.

The recruitment of identified researchers, clinicians, and policy makers (here on referred to as ‘professional’ stakeholders) was through an expression of interest to participate, based on a published communication strategy (www.improvelto.com) [32]. Contact was made by email with study information and up to two reminders. Study information included the project rationale and goals, an outline of the stakeholder groups, the overall study timeframe and anticipated commitment for each round. In the absence of guidelines for the optimal number of Delphi participants, 50 participants were sought with the panel composition based on: the end-user groups anticipated to have most interest in the results; a desire for 20% patient representation [32]; and, diversity in participants’ geographical location and expertise across the hospital-community care continuum. All people who indicated intent to participate were invited to all survey rounds, unless they formally withdrew from the study.

### Delphi surveys

**Fig. 1:**
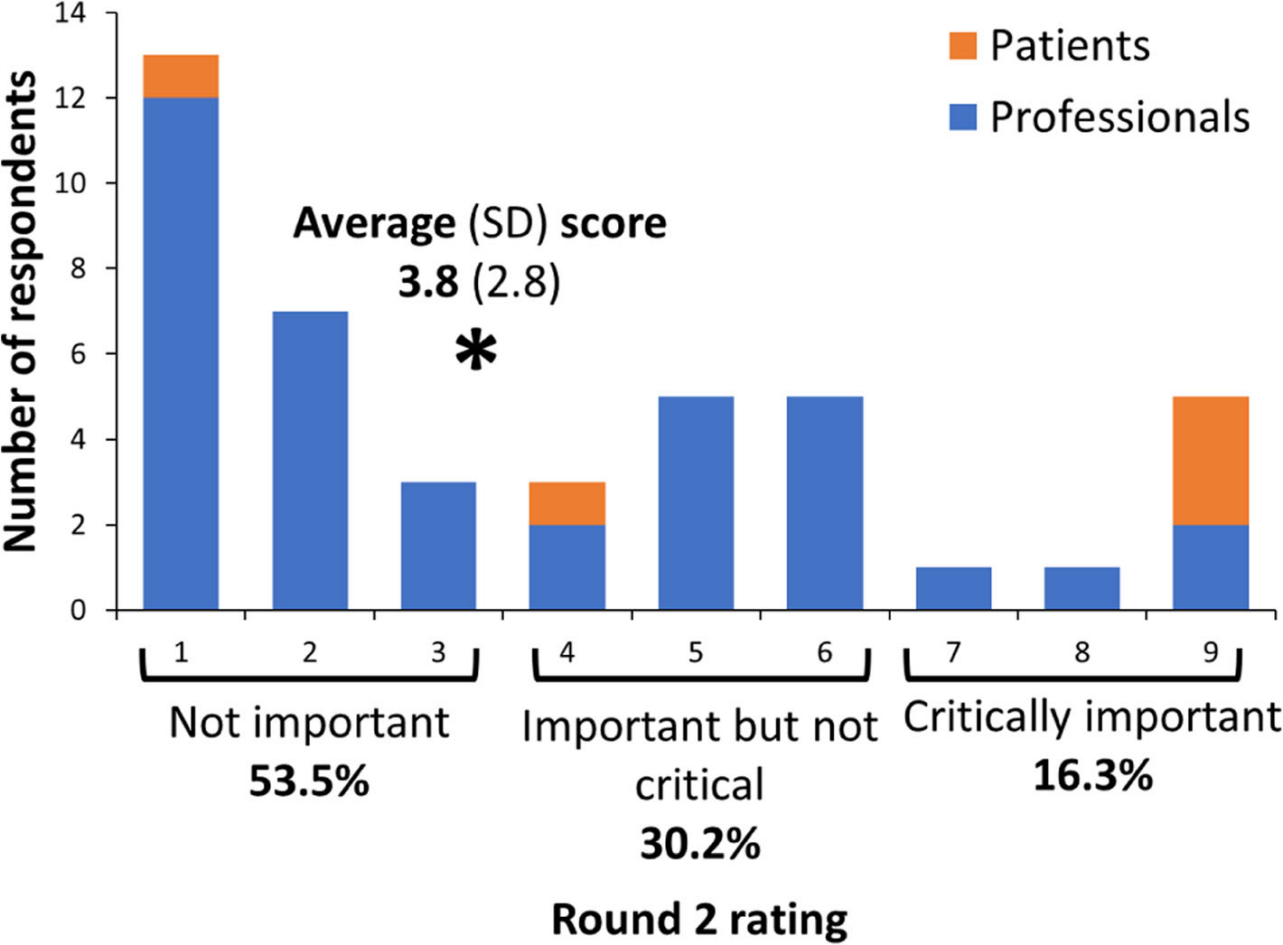
Example histogram, as provided to participants for group level feedback

A maximum of four survey rounds were planned. Each survey was pilot tested prior to distribution by people who were not participants in the Delphi proper; Round 1 was tested by seven multi-disciplinary clinicians and two community dwelling older adults while Rounds 2–4 were tested by three people (an older adult, a clinician and a researcher). Study participants received an electronic survey (www.qualtrics.com) via email and were asked to complete each survey within three weeks, with email reminders at one and two weeks. Participant anonymity was maintained by individualised communication for each round, and a reminder that participants should avoid searching for or seeking opinions of others. After each round, participants were emailed an individual document of their responses. Group level feedback was integrated in Rounds 2 to 4 (Fig. 1).

### Round 1

**Fig. 2:**
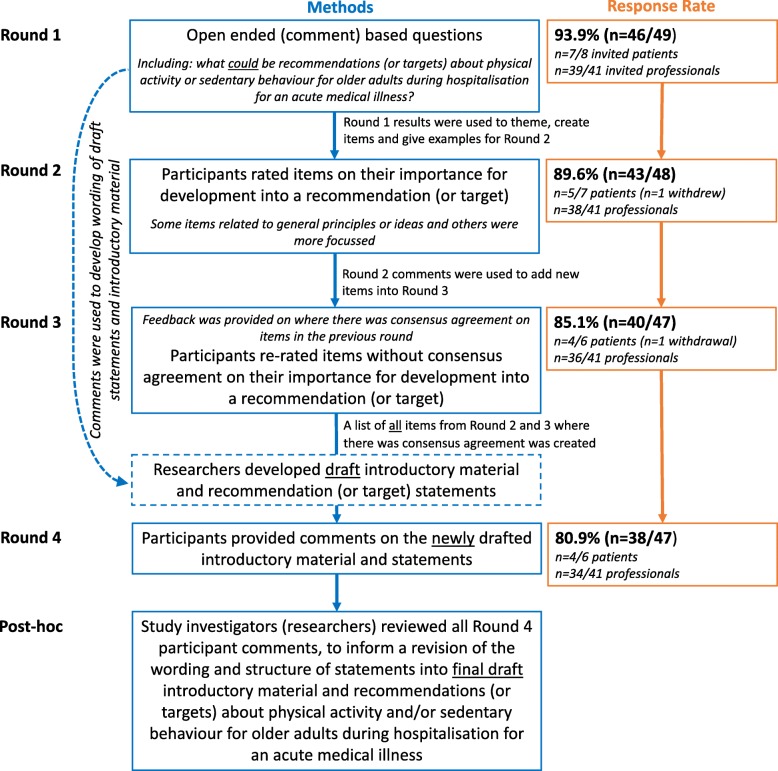
Overview of study procedures and round response rates

Round 1 started in March 2019. Demographic data were collected as relevant to each stakeholder group. All participants were invited to read background information which included: definitions for PA and SB; the World Health Organization’s (WHO) ‘Global Recommendations on Physical Activity for Health: 65 years and above’; [30] and information on how many healthy older Australians are meeting guidelines [6, 43] (participants from other countries were asked to reflect on data from their own country). For the purposes of the study, an ‘acute hospital’ was defined as a place providing 24-h care for people who are unwell and had an unplanned admission, and, ‘acute medical illness’ was deemed not to include ‘elective’ or planned admissions, an admission for which surgery is the main form of treatment, or, admission for a mental health condition. All participants indicated that they understood the background information and definitions. Participants were then asked open-ended questions about older adults while hospitalised with an acute medical illness (here on referred to as hospitalised older adults), including: awareness of any recommendations/targets for PA or minimising SB, what could recommendations/targets be, how patients could achieve suggested recommendations/target, and whether there were conditions or circumstances where suggested targets/aims needed to be modified (Fig. 2). Round 1 responses were qualitatively collated by the lead investigator before confirmation by the research team.

### Round 2

Round 2 was developed based on 16 categories identified from Round 1 analysis, grouped as: PA (*n* = 3 items), SB (*n* = 2 items), people (*n* = 5 items) and organisational factors (*n* = 6 items). Participants were invited to rate each item for the importance with which it should be developed into a recommendation/target (Fig. 2). Scoring was based on the 9-point GRADE scale divided into 3 categories: not important (1–3), important but not critical (4–6), and critical (7–9) [44]. Participants were also asked to rate the applicability of the WHO PA recommendations [30] (and country specific SB guidance) to hospitalised older adults, using a scoring system of not applicable (1–3), somewhat applicable (4–6), and extremely applicable (7–9). Participants could choose to leave items blank (no rating). Consensus agreement was defined *a priori* [32] as: ≥70% of respondents rating an item as ‘critical’ or ‘extremely applicable’ (score ≥ 7) and ≤ 15% of respondents rating an item as ‘not important’ or ‘not applicable’ (score ≤ 3).

### Round 3

Round 3 was developed based on Round 2 analysis (Fig. 2). Participants were invited to re-rate items where consensus agreement was not reached in the previous round, and to rate new items generated from free-text comments in Round 2. Participants were reminded of the study definitions including an explanation of consensus agreement with prompts to give thought to the category labels when rating items (e.g. not important, important but not critical, critically important).

### Round 4

At the completion of Round 3, the research team drafted 29 new statements (Additional file 1, Table S2) with supporting introductory material (Additional file 1, Figure S1), based on participant comments and ratings from the previous three rounds (Fig. 2). All 29 statements on the PA and SB of hospitalised older adults were based on items that reached consensus agreement within Rounds 2–3. In this fourth and final round, participants were asked to comment on or provide alternative wording for each statement, or simply to indicate that they were supportive. Round 4 concluded in October 2019.

### Data analysis

The response rate was determined as the proportion (n, (%)) of panel members to whom the survey was distributed (i.e. signalled intent to participate). Participant characteristics are reported descriptively. For Likert scale items, the mean, standard deviation (SD), median and mean absolute deviation from the median (MAD-M) were calculated using all participant data, and separately for the patient and professional groups. Consensus agreement was calculated based on the number of respondents for each item. The process for creating a final draft of recommendations (or targets) was determined post-hoc. Round 4 verbatim responses for each statement were categorised as either ‘endorsed’ (determined as a positive participant response with wording such as *“agree” “no comment” “supportive” “good”*) or a ‘feedback’ comment (Fig. 2). All feedback comments were reviewed, and revised into succinct statements to create a final draft of recommendations (or targets).

## Results

A total of 59 professionals were sent the study expression of interest, of whom 41 (69.5%) signalled their intent to participate (Additional file 1, Table S1). Out of the 13 patients who completed recruitment screening, eight were confirmed to meet the study eligibility criteria (Additional file 1, Table S1). Therefore, survey Rounds 1 was distributed to 49 people, and patients comprised 16% of the total sample. The participation rate in all 4 rounds was 65.3% (*n* = 32/49) with *n* = 48/49 (98.0%) people responding to at least one round. One patient did not complete any surveys. Demographic characteristics of the 48 people who completed at least one survey are available in Table 1. The response rate for each round is available in Fig. 2.

**Table 1:** Participant characteristics

**Patient stakeholder characteristics**	***n*** **= 7**
Age (years), mean (SD) [range]	70 (6) [65–79]
Highest qualification, n (%)	
PhD	1 (14)
Graduate degree	3 (43)
Secondary school	3 (43)
**Professional stakeholder characteristics**	***n*** **= 41**
Age (years), mean (SD) [range]	47 (9) [28–67]
Residing Continent, n (%)	
Australia	15 (36)
North America	13 (32)
Europe	11 (27)
Asia	2 (5)
Highest qualification, n (%)	
PhD	32 (78)
Masters	2 (5)
Medical doctor	3 (7)
Graduate degree	4 (10)
Professional experience, n (%) ^a^	
Clinician	8 (20)
Clinician + researcher	16 (39)
Researcher	14 (34)
Researcher + guideline developer	3 (7)
Main profession, n ^b^	
Exercise physiologist	6
Medical doctor (physician)	5
Nurse	6
Physiotherapist (Physical Therapist)	22
Public Health physician or scientist	2
Other	3
Years practising in main profession, n (%)	
> 20 years	19 (46)
11–20 years	17 (41)
5–10 years	2 (5)
0–4 years	3 (7)
Main work setting, n (%) ^c^	
Hospital/healthcare facility	9 (22)
University	27 (66)
Other	5 (12)

*SD* standard deviation

^a^participants were asked to indicate what experiences they were drawing on from within the last 10 years, with response options of clinician, researcher, guideline developer and ‘other’; one ‘clinician’ also declared experienced with a national health campaign; one ‘clinician + researcher’ also declared experience as a recent patient and one indicated experience as an educator

^b^participants were able to select more than one profession, three participants declared dual professions so reported numbers do not add up to the sample size of 41; other professions included scientist, academic, behavioural epidemiologist

^c^other responses were a university hospital (*n* = 2), long term care (*n* = 1), research institute (n = 1) and not specified (n = 1)

### Round 1

When participants were asked if they were aware of any recommendations/targets for PA or minimising SB, 12 participants (10 professionals and 2 patients) responded ‘yes’ for PA, and 10 (8 professionals and 2 patients) responded ‘yes’ for minimising SB. Analysis of verbatim responses included reference to 16 publications [17, 30, 39, 45–57]. Some participants expressed an awareness of general guidance or principles, that are not prescriptive on what PA to do in the hospital setting. More broadly, there were two main themes raised in Round 1, firstly the definitions for PA and SB, and secondly, challenges to developing consensus recommendations. Participants suggested that the separation of questions for PA and SB for hospitalised older adults resulted in duplication. For example, one researcher participant commented: “*Minimising sedentary behaviour and increasing physical activity are much the same thing”*. Participants raised questions on whether PA and SB should be considered in isolation or as a continuum, the key reasons given for this query related to how time is substituted between sitting, standing, light activity, moderate to vigorous physical activity (MVPA) and sleep within a 24-h day; whether time spent sitting affects health independent of MVPA in hospitalised older adults; whether sitting should be separated from lying in approaches to SB for hospitalised older adults; whether hospitalised older adults may complete PA in sedentary (sitting/lying) postures (example, chair exercises); and, consideration of energy expenditure for activities, regardless of whether in bed, seated, or standing. Some responses suggested uncertainty as to whether standardised recommendations could be made. An example comment provided by a policy maker participant was: “*While I think a target would potentially be helpful, I’m hesitant as it would potentially be a different target (individualised target) for each patient dependent on ability and amended/updated on an individual basis.”*

### Round 2

When presented with existing WHO guidelines for older adults [30], consensus agreement was reached overall and within both the patient and professional groups that 4 existing recommendations were ‘extremely applicable’ (scored ≥7), and one recommendation was ‘not applicable’ (inverse scoring of ≤3) (Table 2). A greater proportion of patients rated guidelines as ‘extremely applicable’. Optional free-text responses were provided by 23 people. Participant views ranged from suggesting that no guidelines were applicable, to some guidelines being applicable while others not, and, that most apply. The following issues were raised about why consensus may or may not be reached:

- *Focus of guidelines:* participants suggested that the emphasis on cardiovascular, SB, strength and balance guidelines may be different during acute hospitalisations; cardiovascular fitness may be less important; strength and balance components may better relate to the more common problems of hospitalisation; and, sitting may be separated from lying.
- *Approach to endorsing guidelines:* participants identified barriers and practical limitations to meeting potential guidelines; some participants viewed targets as something to still aim for.
- *Issues of dosage:* participants suggested that it may be hard to generalise the dosage of exiting guidelines (intensity, duration or frequency) to hospitalised older adults; the minimum duration of aerobic activity bouts is not included in current Australian [58] or US guidelines [59].
- *Caveats:* participants suggested how changes to wording could alter the applicability.

**Table 2:** Round 2 responses

Item	Mean (SD)	Median (MADM)	Proportion of participants scoring the item ≥7 on the 9-point Likert scale ^a^		
			All *n* = 43	Professionals *n* = 38	Patients *n* = 5
**How applicable are the following recommendations to older medical patients while acutely hospitalised?**					
Older adults should do at least 150 min of moderate-intensity aerobic physical activity throughout the week or do at least 75 min of vigorous-intensity aerobic physical activity throughout the week or an equivalent combination of MVPA	3.8 (2.8)	3 (0.8)	7 (16%)	4 (11%)	3 (60%)
Aerobic activity should be performed in bouts of at least 10 min duration	4.2 (2.8)	4 (0.2)	11 (26%)	8 (21%)	3 (60%)
For additional health benefits, older adults should increase their moderate intensity aerobic physical activity to 300 min per week, or engage in 150 min of vigorous-intensity aerobic physical activity per week, or an equivalent combination of MVPA	2.8 (2.4)	2 (0.8)	4 (9%) ^b^	2 (5%)	2 (40%)
Older adults, with poor mobility, should perform physical activity to enhance balance and prevent falls on 3 or more days per week	6.9 (2.3)	7 (0.1)	27 (63%)	22 (58%)	**5 (100%)**
Muscle-strengthening activities, involving major muscle groups, should be done on 2 or more days a week	6.8 (2.5)	8 (1.2)	27 (63%)	22 (58%)	**5 (100%)**
When older adults cannot do the recommended amounts of physical activity due to health conditions, they should be as physically active as their abilities and conditions allow.	8.5 (1.3)	9 (0.5)	**40 (93%)**	**35 (92%)**	**5 (100%)**
If you can, also try to reduce the time you spend sitting for long periods	7.9 (1.6)	9 (1.1)	**35 (81%)**	**30 (79%)**	**5 (100%)**
All older adults should minimise the amount of time spent being sedentary (sitting) for extended periods	7.6 (1.8)	9 (1.4)	**33 (77%)**	**29 (76%)**	**4 (80%)**
Adults should move more and sit less throughout the day. Some physical activity is better than none. Adults who sit less and do any amount of MVPA gain some health benefits	7.7 (1.7)	8 (0.3)	**34 (79%)**	**29 (76%)**	**5 (100%)**
**Potential recommendations (or targets) for older adults while hospitalised for an acute medical illness. How important is it to develop:**					
***Physical activity***					
1. a general recommendation about physical activity	8.0 (1.7)	9 (1.1)	**38 (88%)**	**33 (87%)**	**5 (100%)**
2. a focussed recommendation about physical activity	6.7 (2.1)	7 (0.3)	28 (65%)	23 (61%)	**5 (100%)**
including the following components					
- frequency (e.g., bouts per day, or, number of days per week)	7.7 (1.4)	8 (0.5)	**37 (86%)**	**32 (84%)**	**5 (100%)**
- duration (e.g., total activity per day)	6.8 (2.0)	7 (0.2)	29 (67%)	25 (66%)	**4 (80%)**
- intensity (e.g., light, moderate, vigorous)	6.3 (2.0)	7 (0.7)	22 (51%)	19 (50%)	3 (60%)
- type (e.g., what activities)	6.8 (1.9)	7 (0.2)	28 (65%)	25 (66%)	3 (60%)
- timing (e.g., when to commence being active during admission, or, what hours of the day)	5.5 (2.8)	6 (0.5)	21 (49%)	18 (47%)	3 (60%)
3. a physical activity recommendation about walking	7.8 (1.2)	8 (0.8)	**38 (88%)**	**34 (90%)**	**4 (80%)**
including the following components					
- that reflects mixed capabilities	8.2 (1.0)	9 (0.8)	**40 (93%)**	**35 (92%)**	**5 (100%)**
- that is tailored to those who can walk independently	7.2 (1.9)	8 (0.8)	29 (67%)	24 (63%)	**5 (100%)**
- that is tailored to those with limited walking capability	7.7 (1.6)	8 (0.3)	**37 (86%)**	**32 (84%)**	**5 (100%)**
- frequency (e.g., number of walks per day, or, number of days per week)	7.4 (1.6)	8 (0.6)	**35 (81%)**	**30 (79%)**	**5 (100%)**
- duration (e.g., how long for)	6.9 (1.9)	7 (0.1)	**31 (72%)**	**27 (71%)**	**4 (80%)**
- target number of steps per day	6.0 (2.4)	7 (1.0)	22 (51%)	18 (48%)	**4 (80%)**
***Sedentary behaviour***					
1. a general recommendation about minimising sedentary behaviour	8.1 (1.2)	9 (0.9)	**39 (91%)**	**34 (90%)**	**5 (100%)**
2. a focussed recommendation about sedentary behaviour	6.8 (2.0)	7 (0.2)	29 (67%)	24 (63%)	**5 (100%)**
including the following components					
- frequency of breaking up time in sitting/lying with standing	7.3 (1.9)	8 (0.7)	**30 (70%)**	26 (68%)	**4 (80%)**
- total sedentary time	6.4 (1.9)	6 (0.4)	21 (49%)	17 (45%)	**4 (80%)**
- sitting out of bed	7.0 (1.8)	7 (0.0)	**30 (70%)**	**27 (71%)**	3 (60%)
***People factors***					
1. a recommendation about the culture, value or philosophy of physical activity in hospital	8.4 (1.0)	9 (0.6)	**41 (95%)**	**36 (95%)**	**5 (100%)**
2. a recommendation about who to engage to change or enable activity in hospital	8.5 (0.7)	9 (0.5)	**42 (98%)**	**37 (97%)**	**5 (100%)**
3. a recommendation about professional roles and responsibilities	8.2 (1.2)	9 (0.8)	**38 (88%)**	**33 (87%)**	**5 (100%)**
including the following components					
- prescription or orders for mobility	7.4 (2.0)	8 (0.6)	**33 (77%)**	**28 (74%)**	**5 (100%)**
- having appropriate assistance (staff) to enable mobility	8.5 (1.0)	9 (0.5)	**40 (93%)**	**35 (92%)**	**5 (100%)**
4. a recommendation about the influence and engagement of patients and relatives	8.1 (1.2)	9 (0.9)	**40 (93%)**	**36 (95%)**	**4 (80%)**
including the following components					
- permissions, guidance or knowledge (where to go and what to do) to enable activity	8.0 (1.2)	8 (0.0)	**38 (88%)**	**35 (92%)**	3 (60%)
- engagement in daily care plans (e.g. timing of doctor visits, meals, observations)	7.7 (1.3)	8 (0.3)	**35 (81%)**	**31 (82%)**	**4 (80%)**
- self-directed, independent or minimally supervised activities	7.9 (1.4)	8 (0.1)	**36 (84%)**	**32 (84%)**	**4 (80%)**
5. a recommendation about staff encouraging patient activity	8.5 (0.8)	9 (0.5)	**41 (95%)**	**36 (95%)**	**5 (100%)**
including the following components					
- encouragement, support, empowerment, or partnership with patients	8.3 (1.0)	9 (0.7)	**39 (91%)**	**34 (90%)**	**5 (100%)**
- self-monitoring and feedback	7.7 (1.3)	8 (0.3)	**35 (81%)**	**31 (82%)**	**4 (80%)**
- daily mobility goal setting	8.0 (1.3)	8 (0.0)	**38 (88%)**	**33 (87%)**	**5 (100%)**
- coaching and application of behaviour change principles	7.7 (1.6)	9 (1.3)	**32 (74%)**	**29 (76%)**	3 (60%)
***Organisational factors***					
1. a recommendation that recognises that a complex issue requires complex solutions	7.8 (1.5)	8 (0.2)	**36 (84%)**	**33 (87%)**	3 (60%)
2. a recommendation about the potential value of policy	7.7 (1.4)	8 (0.4)	**34 (79%)**	**30 (79%)**	**4 (80%)**
3. a recommendation about the potential value of procedures	7.3 (1.4)	7 (0.3)	**30 (70%)**	25 (66%)	**5 (100%)**
4. a recommendation about the potential value of education	7.5 (1.6)	8 (0.5)	**34 (79%)**	**30 (79%)**	**4 (80%)**
5. develop a recommendation about incorporating opportunities for activity into daily care	8.3 (1.2)	9 (0.7)	**42 (98%)**	**37 (97%)**	**5 (100%)**
including the following components					
- focus on function, activities of daily living	8.3 (0.9)	9 (0.7)	**40 (93%)**	**36 (95%)**	**4 (80%)**
- meal-time	7.3 (1.4)	7 (0.3)	**30 (70%)**	26 (68%)	**4 (80%)**
- hygiene (toileting, showering, bathing)	7.8 (1.3)	8 (0.2)	**35 (81%)**	**31 (82%)**	**4 (80%)**
- dressing	7.6 (1.4)	8 (0.4)	**34 (79%)**	**31 (82%)**	3 (60%)
6. a recommendation about the physical environment and resources	8.2 (1.2)	9 (0.8)	**40 (93%)**	**35 (92%)**	**5 (100%)**
including the following components					
- the built environment	7.8 (1.2)	8 (0.2)	**36 (84%)**	**32 (84%)**	**4 (80%)**
- portable adaptations to the environment equipment for activity	8.1 (0.9)	8 (0.1)	**41 (95%)**	**36 (95%)**	**5 (100%)**
- equipment for activity	8.0 (1.2)	8 (0.0)	**38 (88%)**	**34 (90%)**	**4 (80%)**

*MADM* mean absolute deviation from the median, *MVPA* moderate-to-vigorous physical activity, *SD* standard deviation

^a^n (%) results in bold text indicate where consensus agreement was reached

^b^consensus agreement was reached based on inverse scoring, 31 (75%) participants rated the item ≤3 (not applicable)

Participant ratings of potential new recommendations/targets for hospitalised older adults are presented in Table 2. There was consensus agreement that 37 (out of 46) items/concepts were ‘critically important’. There was consensus agreement in both patient and professional groups for 28 items. Within the group of ‘PA’ recommendations, optional free-text responses were provided by 14 participants, expressing issues of: differing guidelines for patients who are dependent versus independent (requiring specificity to context and ability); the balance between broad or general recommendations with focussed recommendations, including risks of these different approaches (recommendations being ignored if too general); evidence for targets; challenges to implementation and methods of measurement; and, coverage of activities other than walking. Within the group of ‘SB’ recommendations, optional free text responses from 11 participants expressed issues of: the balance between general and focussed recommendations, including risks of these different approaches; evidence for focussed targets; challenges to implementation, especially with patients who require assistance to break up SB; consideration of context (when rest is needed for recovery, or SB occurs due to lack of stimulus); and, how sedentary time is broken up and the differentiation of sitting and lying.

Within the group of ‘people’ recommendations, optional free text responses (*n* = 12) expressed issues of: risk culture, communication and the expectations of all about mobility; impacts of the biomedical model (hospital/ward cultures, timing of ward rounds to give free time for fundamental care (meals, mobility), and the limitations of a ‘prescription’ approach for a non-pharmacological intervention; goal setting (including documentation, and opportunities for modification); sensitive staff/patient interactions that are culturally safe and respectful (including to patients with cognitive impairment, an example comment provided by a patient was “*encouragement be seen as 'bullying'”*; and, staff competence, not just availability. Within the group of ‘organisational’ recommendations, optional free-text responses from seven participants expressed issues of: challenges to implementation; interactions and the requirement of multiple components for success; and, evidence for recommendations.

### Round 3

Participant re-rating of potential new recommendations/targets for hospitalised older adults with an acute medical illness resulted in six items reaching consensus agreement as ‘critically important’ (Table 3). When asked about the applicability of existing guidelines for older adults [30], two further guidelines reached consensus agreement as being ‘extremely applicable’ and one recommendation was ‘not applicable’ (inverse scoring of ≤3) (Table 3).

**Table 3:** Round 3 responses

Item	Mean (SD)	Median (MADM)	Proportion of participants scoring the item ≥7 on the 9-point Likert scale ^a^		
			All *n* = 40	Professionals *n* = 36	Patients n = 4
**How applicable are the following recommendations to older medical patients while acutely hospitalised?**					
Older adults should do at least 150 min of moderate-intensity aerobic physical activity throughout the week or do at least 75 min of vigorous-intensity aerobic physical activity throughout the week or an equivalent combination of MVPA	2.7 (2.1)	2 (0.7)	3 (8%) ^b^	1 (3%)	2 (50%)
Aerobic activity should be performed in bouts of at least 10 min duration	3.7 (2.4)	4 (0.4)	5 (13%)	3 (8%)	2 (50%)
Older adults, with poor mobility, should perform physical activity to enhance balance and prevent falls on 3 or more days per week	7.7 (1.5)	8 (0.3)	**34 (85%)**	**31 (86%)**	**3 (75%)**
Muscle-strengthening activities, involving major muscle groups, should be done on 2 or more days a week	7.8 (1.6)	8.5 (0.7)	**33 (83%)**	**30 (83%)**	**3 (75%)**
**Potential recommendations (or targets) for older adults while hospitalised for an acute medical illness. How important is it to develop:**^**c**^					
***Physical activity***			*n* = 39	*n* = 35	n = 4
a focussed recommendation about physical activity	7.4 (1.0)	7 (0.4)	**35 (90%)**	**33 (94%)**	2 (50%)
including the following components					
- duration (e.g., total activity per day)	6.8 (1.7)	7 (0.2)	27 (69%)	**26 (74%)**	1 (25%)
- intensity (e.g., light, moderate, vigorous)	6.4 (1.4)	7 (0.6)	22 (65%)	20 (57%)	2 (50%)
- type (e.g., what activities)	6.9 (1.2)	7 (0.2)	25 (64%)	24 (69%)	1 (25%)
- timing (e.g., when to commence being active during admission, or, what hours of the day)	6.6 (1.7)	7 (0.4)	25 (64%)	22 (63%)	**3 (75%)**
3. a physical activity recommendation about walking should include the following components					
- that is tailored to those who can walk independently	7.7 (1.1)	8 (0.3)	**35 (90%)**	**32 (91%)**	**3 (75%)**
- target number of steps per day	6.7 (1.4)	7 (1.8)	24 (62%)	24 (69%)	0 (0%)
***Sedentary behaviour***					
2. a focussed recommendation about sedentary behaviour	7.7 (1.0)	8 (0.3)	**36 (92%)**	**33 (94%)**	**3 (75%)**
including the following components					
- total sedentary time	6.9 (1.3)	7 (0.2)	26 (68%)	23 (66%)	**3 (75%)**
- types of sedentary breaks ^d^	6.9 (1.7)	7 (0.1)	**28 (72%)**	**26 (74%)**	2 (50%)
- duration of sedentary breaks ^d^	6.7 (1.6)	7 (0.3)	24 (62%)	24 (69%)	0 (0%)
***People factors***					
3. a recommendation about professional roles and responsibilities including the following component					
- ensuring appropriate staff competence to enable mobility ^d^	7.9 (1.2)	8 (0.1)	**34 (87%)**	**32 (91%)**	2 (50%)
5. a recommendation about staff encouraging patient activity including the following component					
- principles of sensitivity and respect (e.g., to culture, physical and mental capability) ^d^	7.8 (1.3)	8 (0.2)	**31 (80%)**	**28 (80%)**	**3 (75%)**

MADM, mean absolute deviation from the median; MVPA, moderate-to-vigorous physical activity; SD, standard deviation

^a^n (%) results in bold text indicate where consensus agreement was reached

^b^consensus agreement was reached based on inverse scoring, 28 (70%) participants rated the item ≤3 (not applicable)

^c^no ‘organisational factors’ were rated in Round 3 as all reached consensus agreement within Round 2, and no new items were generated relating to organisational factors

^d^new items generated from Round 2 responses

### Round 4

**Fig. 3:**
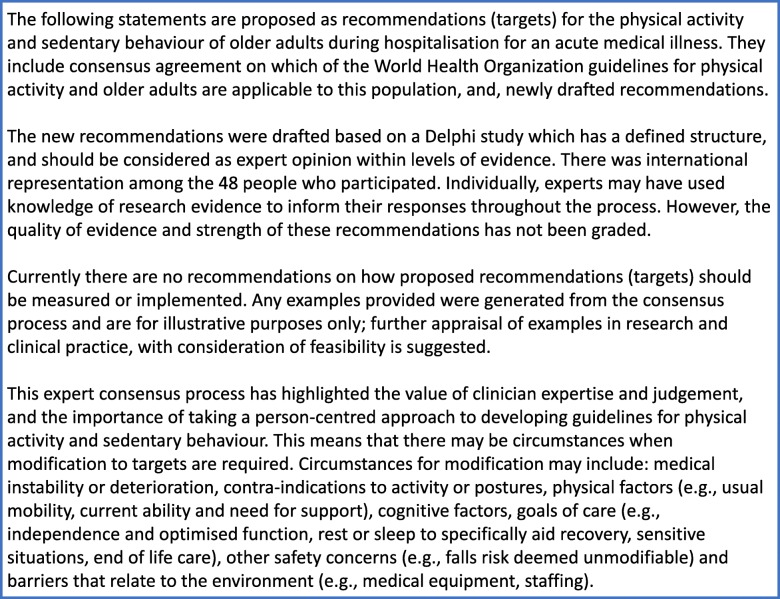
Final draft introductory material (i.e. amended statements based on participant feedback from Round 4)

In Round 4, participants were presented with 29 newly drafted statements under 16 categories, with supporting introductory material (Fig. 2); a summary of the participant responses and draft material are presented in Additional file 1 (Table S2 and Figure S1). Further comments were made by 13 people at the end of the survey (not attached to a particular statement). Comments included positive endorsements (*n* = 6) (e.g., ‘*excellent’ ‘clear and concise’ ‘really good’ ‘I can see how the various stages of the Delphi process have informed these statements’*), suggestions for examples (*n* = 2, stating the need to acknowledge the *‘relative lack of firm evidence’* and/or that statements were *‘too vague’*), that there were too many statements (*n* = 2, overlap with possibility to combine), request for a preamble to explain the context of PA during/throughout waking hours (*n* = 1) and considering the use of both ‘recommendations’ and ‘targets’ terminology (*n* = 1). In the post hoc analysis, there was ‘endorsement’ from ≥70% of respondents for 12 of the 29 newly drafted statements. Based on respondent ‘feedback’ comments within Round 4, a final draft of introductory material (Fig. 3) and recommendations (or targets) (Table 4) were created.

**Table 4:** Round 4 final draft recommendation/targets

Final draft recommendations for physical activity and sedentary behaviour of older adults while hospitalised with an acute medical illness		Source of recommendation
**Existing recommendations**	The following recommended levels of physical activity for adults aged 65 and above remain applicable while hospitalised with an acute medical illness:	
	• When older adults cannot do the recommended amounts of physical activity due to health conditions, they should be as physically active as their abilities and conditions allow.	WHO guidelines [30]
	• Older adults, with poor mobility, should perform physical activity to enhance balance and prevent falls on 3 or more days per week.	WHO guidelines [30]
	• Muscle-strengthening activities, involving major muscle groups, should be done on 2 or more days a week.	WHO guidelines [30]
	Alternative wording for the first recommendation above may be applicable for the context of older adults while hospitalised with an acute medical illness:	
	• When older adults cannot do the recommended physical activity due to illness or health conditions, they should be as physically active as their abilities and health status allows. ^b^	Item 1c ^b^ (Table S2)
	Consistent with country specific guidance for older adults, the following recommendations have a component specific to sedentary behaviour that remains applicable while hospitalised with an acute medial illness:	
	• adults should move more and sit less throughout the day*,* some physical activity is better than none,^b^ and adults who sit less and do any amount of MVPA gain some health benefits.	US guidelines [59] Item 1b^b^ (Table S2)
	• all older adults should minimise the amount of time spent being sedentary (sitting) for extended periods.	UK guidelines [60] ^a^
	• if you can, also try to reduce the time you spend sitting for long periods.	AUS guidelines [39]
**Newly developed recommendations**	There are some overarching principles that are relevant to all of the newly developed recommendations (or targets), relating to people and organisational factors as follows:	
	• A person-centred approach should be taken to engage and enable older adults to be physically active and minimise sedentary behaviour during hospitalisation.^b^	Item 9a^b^ (Table S2)
	• Enabling physical activity and minimising sedentary behaviour in hospital should be a shared responsibility; all health care professionals, people at different organisational levels, caregivers and relatives, volunteers, and older adults have abilities to contribute.	Item 7^b^ (Table S2)
	• When encouraging physical activity and minimising sedentary behaviour, people should: o act with sensitivity and respect by partnering with, supporting and being ready to hear the perspective of older adults.^b^ o be culturally responsive and mindful of older adults’ physical and mental capabilities.^b^	Items 10a^b^ and 10b^b^ (Table S2)
	• Opportunities for physical activity and minimising sedentary behaviour should be incorporated into the daily care of older adults with a focus on function, independence and activities of daily living.^b^	Item 15a^b^ (Table S2)
	**Physical activity** (defined as, any bodily movement produced by skeletal muscles that requires energy expenditure) [2]	
	• Older adults should aim to be as active as possible during hospitalisation for an acute medical illness, adding movement into everyday activities and incrementally if required.	Item 1a (Table S2)
	• Physical activity should be accumulated regularly, in bouts throughout the day.	Item 2 (Table S2)
	• Walking is one example of physical activity for older adults while hospitalised.	Item 3a (Table S2)
	• For older adults who are able, walking should be accumulated regularly throughout the day and for progressively longer periods.	Items 3d and 3e (Table S2)
	• Older adults who can walk independently should be encouraged to do so, considering their current and usual ability.^b^	Item 3b^b^ (Table S2)
	• Older adults who require help to walk should be assisted, considering their current and usual ability.	Item 3c (Table S2)
	• Other types of activity should be considered for people who are unable to walk.	Item 3a (Table S2)
	**Sedentary behaviour** (defined as, the waking time spent sitting or lying down, or, according to an energy expenditure threshold of ≤1.5 METs) [10]	
	• Older adults should aim to minimise long periods of uninterrupted sedentary behaviour during waking hours while hospitalised.	Item 4 (Table S2)
	• When possible, sitting out of bed and movement from bed to chair are preferable to time spent lying in bed.	Item 5a (Table S2)
	• Older adults should break up sedentary time by standing up and or/walking as often as possible, with assistance as needed; a modifiable target may be to stand up and/or walk each waking hour.	Item 5b (Table S2)
	• If standing up is not possible, a modifiable target may be completing light intensity movements in a seated or lying position.	Item 5c (Table S2)
	The following recommendations related to **people factors** that may support physical activity and minimising sedentary behaviour:	
	• To address physical activity and sedentary behaviour during hospitalisation, the culture, philosophy of care, and value of physical activity in hospitals should be examined.	Item 6 (Table S2)
	• Clear professional roles and responsibilities are needed to enable older adults to be physically active and minimise sedentary behaviour; this may include directives for mobility and having appropriately trained people who are available to assist older adults.	Item 8 (Table S2)
	• When enabling older adults to be physically active and minimise sedentary behaviour, consideration should be given to what permissions for activity, instructions (including self-directed, independent or minimally supervised activities), inclusion of caregivers and knowledge of the environment and daily care plans is required.	Item 9b (Table S2)
	• Principles of behaviour change including mobility goal setting, self-monitoring and feedback may support physical activity and sedentary behaviour in the acute hospital setting.^b^	Item 10c^b^ (Table S2)
	The following recommendations relate to **organisational factors** that may support physical activity and minimising sedentary behavior:	
	• Consideration should be given to moments for physical activity and minimising sedentary behaviour as part of common care activities like mealtime, hygiene and dressing.^b^	Item 15b^b^ (Table S2)
	• Consideration should be given to the value of education and training as it relates to the shared responsibility of enabling physical activity and minimising sedentary behaviour in hospital (e.g. older adults, caregivers and relatives, developing and practicing health care professionals, people at different organisational levels).^b^	Item 14^b^ (Table S2)
	• To address physical inactivity and sedentary behaviour during acute hospitalisation, it is important to understand the complexity of local issues and consider hospital-system based solutions that address the physical and social environment, along with other factors. o consideration should be given to the potential value of policies and procedures; it may be relevant to address, ▪ roles and responsibilities ▪ work organisation (such as transport and bed allocations) ▪ adverse event reporting ▪ care plans and ward rounds ▪ methods for prompting behaviours of older adults and health care professionals ▪ the use of digital technologies or devices for monitoring or assistance	Items, 11, 12, and 13 (Table S2)
	• Consideration should be given to the influence of the physical environment on the ability for older adults to be active, including in- and out-door environments, portable adaptations and equipment.^b^	Item 16a and 16b^b^ (Table S2)

*AUS* Australian, *MET* metabolic equivalent of task, *MVPA* moderate-to-vigorous physical activity, *UK* United Kingdom, *US* United States, *WHO* World Health Organization

^a^Since conduct of this Delphi study, UK guidance has been replaced with a newer version of recommendations [41] that were not tested for consensus agreement on applicability to hospitalised older adults

^b^denotes recommendations/targets that were revised from original draft statements in Round 4 that received responses of endorsement from ≥70% of respondents

## Discussion

This study provides the first international consensus for recommendations on PA and SB for older adults while hospitalised with an acute medical illness. The main messages from consensus agreement on the applicability of existing PA/SB guidelines to this inpatient population are that hospitalised older adults should: be as physically active as their abilities and condition allows [30]; minimise time spent sitting or sedentary for extended periods [40]; and, move more and sit less throughout the day [59]. Muscle strengthening and balance exercises were also viewed as important, although participant responses suggested that strength and balance activities may be performed more frequently (daily) by hospitalised older adults, than stated in current guidelines. The wording of the newly drafted PA and SB recommendations for hospitalised older adults was such that some were very general statements and others were more focussed. The newly drafted recommendations begin to address contextual issues such as the influence of people and organisational factors. Focussed recommendations such as those on PA or sedentary break frequency did not reach consensus agreement early on and required development over several survey rounds.

This study recognises the problems of inactivity in hospitals around the world [9] and responds to a need for recommendations specific to hospitalised older adults. This study centred on inpatients with a medical illness to be consistent with other studies [16, 17, 19] and balance the internal validity of recommendations to the clinical population (specificity) without being too restrictive (recommendation generalisability). Patients with surgical admissions can similarly experience low PA (step counts) [61] and SB [62], but may be better supported with post-operative mobility or ERAS protocols [29]. Still, all patients in hospital are exposed to system constraints, such that these study findings may have broader applications.

The newly drafted recommendations build on the principle that some activity is better than no activity by starting to provide targeted guidance on breaking up sedentary time (Table 4, source items 5a-c as per Table S2) and an individualised approach to PA with light and variable intensity activity options for hospitalised patients like walking (Table 4, source items 3a-c as per Table S2). To progress this guidance further, research in the hospital setting is needed to understand the dose-response relationship of PA to clinical outcomes, and potential modulation of the effects of prolonged SB by PA for older adults with an acute medical illness. Even in the general population, there are challenges to making SB guidelines such as on the frequency of breaks [63]. For populations with limited mobility and acute illness, differentiating sitting from lying has short term physiological effects including improved pulmonary function (for example lung volumes) [64] and stimulation of an exercise response (for example increased oxygen consumption, minute ventilation, mean arterial pressure and heart rate) [65]; but more research is required to underpin SB recommendations in acute populations and impacts on other patient centred outcomes.

Potential risks of increased PA in acutely unwell populations need to be monitored and managed. An increased risk of falls is often cited as a reason to avoid increasing PA in hospitalised older adults, however this is not supported by evidence and existing fall prevention strategies such as bed/chair alarms combined with a punitive staff culture may accentuate SB and functional decline, thereby increasing falls risk [66]. For hospitalised older adults, delirium prevention programs like the ‘Hospital-Elder-Life-Program’ (HELP) are highly effective at reducing the odds of falls [27]. A key strategy for fall prevention, HELP and similar programs [26, 67] is patient mobility and exercise. Expert consensus guidelines have been used to support safe early mobility for the most critically unwell patients [68], alongside reviews of adverse event data from exercise-based trials [69], such that a similar approach may progress changes in practice for patients admitted to general wards.

In a complex setting like a hospital, use of a systems framework may be helpful to understand SB and how to influence practice [20]. The recommendations from this study align with operationalisation of the systems of sedentary behaviour framework [9]. Furthermore, the ‘people’ (and culture) recommendations are grounded in qualitative research perspectives from patients, who have expressed a desire for purposeful activity and autonomy, assistance for out-of-bed activity, a balance with physical and mental rest, and understanding of sources of inactivity in the hospital environment and work practices [70–72]. Research with health professions suggests instances of incorrect assumptions about patient motivation [72] and descriptions of a complex social context where behaviours are shaped by professional identities and blurring of responsibilities [73, 74]. All professional groups have expertise that can promote patient mobility and improve outcomes [75]. While physiotherapists’ have particular expertise in mobility issues and are skilled to provide PA counselling on staying active in hospital [76, 77], there are examples of nursing-led, function focussed care programs [25] that consider how patients spend their time outside of any physical therapy sessions, and medical champions within multi-disciplinary programs [26, 27, 75].

This study has several methodological strengths, including representation of a range of stakeholder groups, even though the proportion of included patients in the Delphi (16%) was slightly under the targeted 20%. Stakeholder and particularly patient involvement with greater diversity will be important for future work and implementation. The engagement of participants was strong as evidenced by recruitment, the response rates to each round (and items within rounds), and qualitative comments in support of the project. An example comment provided by a clinician participant was *‘I am impressed with how this research has targeted and progressed on what I think is a very crucial topic, and hope it gets more traction and influence.’* Methodological rigor included using an a priori definition for consensus and item rating system that has been used in other studies and ensured an item does not achieve consensus if there were a strength of opposing views. There were no items in the present study that failed to reach consensus (i.e. otherwise achieved a rating of ‘critically important’ by ≥70% of respondents) because of ≥15% of respondents also rated the item ‘not important’. However, there were some occasions where patient participants indicated they felt unqualified to answer particular questions. Each survey was pilot tested, but separate surveys or questions for different stakeholder groups could have been used [78].

Limitations of the study include progression to the point of final draft recommendations only. Recommendations that are both practicable and robust in the interpretation of an evidence base can be achieved with the GRADE approach. However, it was not appropriate to use GRADE for the present study as the authors perceived the evidence base to be insufficient, with a predominance of observational rather than interventional studies [11]. Therefore, the authors advise judicious use of these draft recommendations and acknowledge that both appraisal of existing evidence and generation of new evidence is required to support recommendations. The draft recommendations may be adapted and finalised by convening an expert working group, and with end-user validation they may be supplemented by strategies for implementation. In order to inform future interventions studies and develop the evidence base itself, agreement on the crucial outcomes that should be measured in research studies of PA and SB during hospitalisation is required.

While a strength of the study was recruitment of international participants, the panel was mostly from Western countries that rank highly on the human development index, which may limit the generalisability of the draft recommendations. For pragmatic reasons, patient stakeholders were not sought from countries other than Australia. The inclusion of researcher and policy maker stakeholders increased the diversity of professional backgrounds represented in the panel, but the most highly represented group was Australian physiotherapists (clinicians and researchers), which likely influenced the perspectives of the recommendations. The study may have been strengthened by including patients from other countries and other professional groups with an interest in patient activity.

## Conclusions

This study generated a range of newly drafted recommendations on PA and SB for hospitalised older adults with an acute medical illness and is a contemporary reflection of the expert thinking of researchers, multi-disciplinary clinicians, policy makers and patients. Older adults’ PA and SB during hospitalisation should be in line with some current recommendations, with the following overarching principles (that reached the highest degree of consensus): acting with respect and person-centredness when working with older adults; being responsive to peoples culture and their physical and mental capabilities; incorporating PA throughout daily care with a focus on function and activities of daily living; and sharing the responsibility of enabling PA and minimising SB. These recommendations may guide future research priorities and co-designed clinical trials. Implementation with the development of supporting resources is also required.

## Supplementary information

**Additional file 1: Table S1:** Expert Panel Recruitment by Stakeholder Group. **Table S2:** Draft recommendation/targets as presented to participants for feedback in Round 4 and summary of participant responses. **Figure S1:** Draft introductory material as presented to participants for feedback in Round 4.

## Data Availability

The datasets used and/or analysed during the current study are available from the corresponding author on reasonable request.

## Abbreviations

CREDES: Recommendations for the Conducting and REporting of DElphi Studies
COMET: Core Outcome Measures in Effectiveness Trials
ERAS: Enhanced Recovery After Surgery
GRADE: Grading of Recommendations Assessment, Development and Evaluation
MAD-M: Mean Absolute Deviation from the Median
MET: Metabolic Equivalent of Task
MVPA: Moderate-Vigorous Physical Activity
PA: Physical Activity
SB: Sedentary Behaviour
SD: Standard Deviation
WHO: World Health Organization
