# Supplementary material for: Recommendations for older adults’ physical activity and sedentary behaviour during hospitalisation for an acute medical illness: an international Delphi study
Source: Int J Behav Nutr Phys Act. 2020 May 25;17:69. doi: 10.1186/s12966-020-00970-3 (PMC7249667; doi:10.1186/s12966-020-00970-3)
Supplement: Supplementary file 1 — Additional file 1: Table S1: Expert Panel Recruitment by Stakeholder Group. Table S2: Draft recommendation/targets as presented to participants for feedback in Round 4 and summary of participant responses. Figure S1: Draft introductory material as presented to participants for feedback in Round 4. [file 12966_2020_970_MOESM1_ESM.docx]

**Additional File 1 (supplementary material)**

**Table S1:** Expert Panel Recruitment by Stakeholder Group

| **Stakeholder group** | **How panel members were recruited** | **Target sample size** | **No. invited ^a^** | **No. agreed to participate (% participation rate)** |
| --- | --- | --- | --- | --- |
| **Clinical Researchers**: from 10 different countries (Australia, Belgium, Brazil, Canada, Denmark, Israel, Japan, Norway, United Kingdom, United States) | Step 1: the research team selected four systematic reviews ^1-4^ ^b^ and compiled a list of all corresponding authors from studies within each review that used an accelerometer-derived measure of PA and/or SB in a group of older adults during hospitalisation for an acute medical illness; the corresponding author of each systematic review was also contacted ^2-4^ | 20 | 24 | 12 (50%) |
|  | Step 2: the research team purposefully invited individual clinical researchers (Australian and international) for their expertise in PA and/or SB with particular conditions that may result in an acute medical hospital admission, being respiratory disease (COPD), cardiac disease (heart failure), cancer, and stroke. |  | 10 | 6 (60%) |
|  | Step 3: the research team reviewed all recommendations of other potential participants, as suggested by invited panel members who were contacted as part of Step 1 and Step 2 above (whether they accepted or declined the invitation to participate); the research team reviewed the publicly available profile of each suggested participant, and prioritised further invitations based on maximising the location and expertise diversity of the panel. |  | 6 (of 47 suggestions) | 4 (67%) |
| **Clinicians/Professional Associations** from Australia, UK, Canada and USA | The research team contacted national professional associations that represent physiotherapists, medical doctors (physicians), and nurses, in Australia, the UK, Canada and the USA, asking each to identify one representative to participate ^c^ | 12 | 12 | 10 (83%) |
| **Policy makers** from Australia, Canada, United Kingdom and United States | The research team selected and contacted an academic representative from the guideline development teams for the published National PA and/or SB guidelines for older adults from Australia, the UK, Canada and the USA. | 7 | 4 | 4 (100%) |
|  | The research team contacted international societies that advocate for improving PA reducing SB, ^d^ and requested each to nominate one person to participate in the study. Where more than one representative was nominated and interested to participate, the research team accepted their participation |  | 3 | 5 (167%) |
| **Patients** from Australia | The research team approached the ‘Health Consumers Alliance of South Australia’ to distribute recruitment materials; an online facebook advertisement was run, targeting South Australian’s aged ≥65 years over a 3-week period | 20% of final sample | 13 complete responses to advertising (of 53 site hits) | 8 (62%) |

^a^ five of the invited clinical researchers declined (four cited workload/capacity at the time, one was agreeable but suggested an alternative individual with expertise more closely aligned to the study who was invited in place)

^b^ corresponding authors from eligible studies that were identified though a preliminary search for a (unpublished) scoping review by the lead author were also included.

^c^ the associations were:

- Australia: Australian Physiotherapy Association (targeting gerontology and cardiorespiratory groups); Australian College of Nursing (targeting the healthy ageing community of interest); Royal Australasian College of Physicians (targeting the Adult Medicine Division, General and Acute)
- UK: Chartered Society of Physiotherapy (targeting respiratory care and working with older people associations); Royal College of Nursing (targeting older people network); initially contacted the Society for Acute Medicine (UK), but after no reply contacted British Geriatric Society
- Canada: Canadian Physiotherapy Association (targeting seniors health and cardiorespiratory divisions); Canadian Nurses Association (targeting acute care nurse practitioners); Canadian Society of Internal Medicine (no representative provided)
- USA: Initially contacted the American Physical Therapy Association (targeting geriatric and acute sections) but after no reply contacted the Johns Hopkins Activity and Mobility Promotion group; American Nurses Association (targeting Adult-Gerontology Acute Care Nurse Practitioner group); American College of Physicians (targeting internal medicine/hospitalists; no representative provided)

^d^ the organisations were: the International Society of Behavioural Nutrition and Physical Activity (ISBNPA); the International Society for Physical Activity and Health (ISPAH); and the Australian, Physiotherapists for Physical Activity (Physios4PA). ISPAH and Physios4PA nominated two people, both of whom were accepted for participation. One person dually represented policy maker and clinical researcher categories.

**Table S2:** Draft recommendation/targets as presented to participants for feedback in Round 4 and summary of participant responses

| **Endorsement n (% of item respondents) ^a^** | **Summary of comments** |
| --- | --- |
| ***Introductory material*** | |
| n = 22 ^b^ (64.7%) | n=12  **Methods detail:** include mention that the Delphi has a defined structure/process, number of participants, international panel  **Other inclusions:** acknowledge the WHO guidelines for community but not hospital settings; who the targets are for; role of clinician expertise and judgement; define person-centred approach  **Wording:** revise first sentence, wording of ‘rest to aid recovery’ ‘further appraisal of examples’ and ‘falls’, clarify hierarchy of evidence |
| ***Physical activity*** | |
| 1a) Older adults should aim to be as active as possible, building movement into every day during hospitalisation for an acute medical illness. | |
| n=24 (68.6%) | n=11  **Wording:** utilise opportunities for movement throughout the waking hours of every day; incorporate physical activity in everyday routines; unsure about "building movement into every day" instead "adding movement into their day at every opportunity"; increasing activity and mobility every day  **Specificity of the recommendation:** suggestions for guidance on frequency, step/distance target, being incremental, not necessarily back to baseline  **Audience for the recommendation:** wording suggests onus on the patient; should be for health professionals/staff too  **Coverage of concepts:** movement a little too "sedentary", include everyday activities |
| 1b) Some physical activity is better than no activity. | |
| n=28 (80%) | n=7  **Wording:** better than no activity at all; even just sitting out of bed is likely to be beneficial; some is good, more is better; bed/chair activity if mobility is limited  **Coverage of concepts:** presuming patients can do something; statement does not underline the importance of activity  **Example:** should be followed by an example |
| 1c) When older adults cannot do the recommended physical activity due to illness or health conditions, they should be as physically active as their abilities and health status allows. | |
| n=26 (74%) | n=9  **Wording:** needs rephasing; use it or lose it; older adults need to do as much as they can.  **Example:** add examples here  **Coverage of concepts:** not clear what the "recommended PA” is during acute illness  **Structure of recommendation:** link this statement to *[recommendation 2]* e.g. 'Specifically activity should...'  **Interaction with other concepts:** similar to statement 1a  **Audience for the recommendation:** unsure if audience will know what recommended physical activity levels |
| 2) Physical activity should be achieved in frequent bouts. | |
| n=9 (25.7%) | n=26  **Wording:** unclear what is meant by frequent; add across/during/throughout the day; regular basis or through waking hours; confusing or vague  **Coverage of concepts:** more guidance should be provide regarding "frequent"; unsure if addressing both increased PA and reduced SB with this  **Specificity of the recommendation:** indicate times per day/per week; minimum bout time or an example e.g. at least hourly or walking 2-3x  **Scope for personalised modification:** listen to the body; stay as active as possible; short bouts  **Audience for the recommendation:** wording may not be clear to older adults |
| 3a) Walking is one example of physical activity for older adults while hospitalised but other types of activity should be considered for people who are unable to walk. | |
| n=16 ^c^ (44.4%) | n=20  **Example:** have an example of an activity for those who can't walk  **Coverage of concept:** bed and chair exercises, gentle strengthening  **Wording:** add "regular walking"; aiming for out of bed activities where possible.  **Structure of recommendation:** possibly would be better split into two clear statements  **Scope for personalised modification:** consider if state something about limitations for people with significant balance issues |
| 3b) Older adults who can walk independently should be encouraged to do so, considering their usual and current ability. | |
| n=27 (77.1%) | n=8  **Wording:** add "by all health care professionals"; consider usual and current ability; be enabled/encouraged to; regularly  **Example:** illustrate with an example  **Coverage of concepts:** add a general statement about the importance of mobility even for sick people  **Scope for personalised modification:** patients should not be made to feel inadequate in comparison to others if they cannot do this |
| 3c) Older adults who require help to walk should be assisted, considering their usual and current ability. | |
| n=24 (68.6%) | n=11  **Wording:** revise wording of current and usual ability; focus more on restoring/maintaining/rehabilitating previous function and improving function where feasible; clarify "assisted"  **Example:** expand with suggestions of the types of assistance that could be given, e.g. walking aids or physical assistance and by who  **Coverage of concepts:** statement is too obvious; include patient carers *[who should provide the assistance]*  **Scope for personalised modification:** there may be limitations to staffing, ward design, paperwork, and other barriers; reduce the fear factor  **Interaction with other concepts:** agree but *[statement 3a]* is an 'out' |
| 3d) For older adults who are able, walking should be incorporated daily and may be achieved in frequent bouts. | |
| n=17 ^b^ (50%) | n=17  **Wording:** rephrase frequent bouts; episodes or occasions; regularly throughout/across the day; encouragement  **Specificity of the recommendation:** vague terminology; unclear what is meant by frequent  **Interaction with other concepts:** *[statement 3e]* covers frequency and supersedes this recommendation; combine *[statements 3e and 3d]*  **Coverage of concept:** emphasise regularly of walking  **Scope for personalised modification:** consider if state something about limitations for people with significant balance issues |
| *3e) Older adults who are able to walk, should walk at least 2-3 times per day and for progressively longer periods, or be as active as possible.* | |
| n=17 (48.6%) | n=18  **Coverage of concepts:** lots in this sentence; final six words aren't helpful/unnecessary; good as a quantifiable instruction is given  **Overlap/similarity to other concepts:** combine *[statements 3e and 3d];* prefer *[statement 3e]*; appear to suggest different recommendations *[statements 3e and 3d]*  **Specificity of the recommendation:** suggest minimum 2-3 times per day; suggest at least 4-5 times per day; unclear where 2-3 times comes from  **Wording:** include throughout rather than per day; vague statement |
| ***Sedentary behaviour*** | |
| 4) Older adults should aim to minimise long periods of sedentary behaviour while hospitalised. | |
| n=23 (65.7%) | n=12  **Wording:** additions including "During waking hours” “support” or “if possible” or “uninterrupted SB”  **Coverage of concepts:** deconditioning starts within 24 hours of admission; benefits of the recommendation; requires carer/nursing assistance  **Specificity of the recommendation:** unclear what is a long period  **Audience for the recommendation:** wording may not be clear to older adults  **Definitions:** define sedentary time first |
| 5a) While *sitting and lying down are sedentary postures, sitting out of bed should be considered preferable to time spent lying in bed.* | |
| n=21 (60%) | n=14  **Wording:** consider removing first part or revising the start; add “when possible”  **Specificity of the recommendation:** choice of the word considering (which is not ‘doing’ things); uncertain about phrasing (indirect)  **Coverage of concepts:** agree with concept (when ceiling of patient function, involves some transitions); not exercise unless moving bed to chair; lack of evidence; exercises in sitting; standing is a sedentary posture too |
| 5b) Older adults should stand up as often as possible, with assistance as needed; a modifiable target may be to stand up each waking hour. | |
| n=22 (62.9%) | n=13  **Wording:** would suggest adding "walking" or across the day; unclear what modifiable target means  **Specificity of the recommendation:** include more prescription; how long to stand for; uncertain about phrasing (indirect)  **Interaction with other concepts:** potential to link with *[statement 4]*; unclear if the desire is just to stand every hour or walk/move as well; *[statement 5c]* may be enough and cover this point  **Scope for personalised modification:** may be relevant for those who cannot ambulate; may depend on pain levels  **Coverage of concepts:** mention walking here as well; older adult should stand up and walk when possible; assistance is not always available |
| *5c) Older adults should aim to break up sedentary time in a standing position but if not possible, a modifiable target may be completing light intensity movements in a seated or lying position.* | |
| n=19 (54.3%) | n=16  **Wording:** include "in a standing or walking position"; statement implies standing is a SB, comment on regularity define frequency of 'breaking up', vague statement; unclear what modifiable target means  **Example:** examples might be helpful (alternatives to standing, light intensity movement)  **Interaction with other concepts:** maybe combine with *[statement 5b]*  **Coverage of concepts:** uncertain about phrasing and meaning (convoluted); should it be in an upright posture preferably displacing with movement over static standing |
| ***People factors*** | |
| 6) To address physical activity and sedentary behaviour during hospitalisation, the culture, philosophy of care, and value of activity in hospitals should be considered. | |
| n=23 ^c^ (63.9%) | n=13  **Wording:** use examined rather than considered; wording "the value of activity" might be ambiguous; add “physical” before “activity”; activity should be part of the culture and philosophy of care; vague statement/meaning not clear  **Issues of implementation:** requires a whole needs approach by qualified staff; job prescriptions and staffing levels  **Example:** on how this operationalized or what it means in practical sense  **Coverage of concepts:** mention the stakeholders who need to be on board and what kinds of change might be needed (e.g. co-designed policies)  **Interaction with other concepts:** link this statement to the specific recommendations below |
| *7) Enabling physical activity and minimising sedentary behaviour in hospital should be the shared responsibility of all health care professionals, people at different organisational levels, caregivers and relatives, volunteers, and older adults in a person-centred approach.* | |
| n=29 ^c^ (80.6%) | n=7  **Issues of implementation;** dissemination in patient rooms; awareness and prevention campaigns  **Coverage of concepts:** best left to qualified staff; statement could be interpreted in many ways leading to inappropriate people giving PA advice; society responsibility for promoting these behaviours  **Structure of recommendation:** long statement (break up or remove last part)  **Wording:** define "person-centred" |
| 8) Clear professional roles and responsibilities are needed to enable older adults to be physically active and minimise sedentary behaviour; this may include medical direction for mobility and having appropriately trained people who are available to assist older adults. | |
| n=20 (57.1%) | n=15  **Coverage of concepts:** include which appropriately trained staff, in making people aware they have skills to help; every team members' responsibility, include family caregivers; not sure/don’t agree with medical direction/prescription (query if most suited profession and not assessed function) or limiting to medical (include physiotherapy occupational therapy, nursing)  **Interaction with other recommendations:** use this and modify *[statement* 7*];* remove this statement; mention of doctors in conflict with *[statement 7]* everyone’s responsibility  **Wording:** emphasise that one visit from 'the physio' is hardly adequate’ or 'all professional staff have a role to play’; add 'defined roles for different members of the MDT'; clarify ‘medical direction’  **Issues of implementation:** appropriately trained people; available and accessible equipment; resources links culture and staffing |
| *9a) A person-centred approach should be taken to engage and enable older adults to be physically active and minimise sedentary behaviour during hospitalisation.* | |
| n=29 (82.9%) | n=6  **Wording:** not clear; review choice of language; define/operationalise "person-centred"  **Example:** some specific guidance might help |
| *9b) When enabling older adults to be physically active and minimise sedentary behaviour, consideration should be given to any required permissions for activity, guidance (including self-directed, independent or minimally supervised activities), knowledge of the environment, enactment of daily care plans, and the inclusion of caregivers.* | |
| n=22 (62.9%) | n=13  **Wording:** a long/wordy statement (but seems relevant for inclusion); clarify what is meant by “guidance” and “required permissions”  **Coverage of concepts:** consider who gives permission for activity; permissions for activities the exceptions (activity the default unless stated by the physician)  **Issues of implementation:** not clear how this works; patient refusal to exercise; therapy plan can be implemented by others |
| *10a) When encouraging physical activity and minimising sedentary behaviour, people should act with sensitivity and respect by partnering with, supporting and being ready to hear the perspective of older adults.* | |
| n=26 (74.3%) | n=9  **Wording:** include the words motivating choices of older adults ; revise wording (too convoluted); health professional rather than people; unsure if wording meets the objective of a recommendation about staff encouraging patient activity  **Issues of implementation:** good to be person centred, collaborative goal setting makes targets more achievable; changes with ageing  **Structure of recommendation:** consider dividing into separate statements with more detail for each |
| *10b) When encouraging physical activity and minimising sedentary behaviour, people should be culturally responsive and mindful of older adults' physical and mental capabilities.* | |
| n=30 (85.7%) | n=5  **Coverage of concepts:** nice inclusion of ethnicity, the individual, cognition and social/environmental factors; limitations of the illness itself  **Wording:** choice of wording, evaluate rather than mindful, cognitive rather than mental; health professional rather than people  **Issues of implementation:** difficult with time constraints in hospitals and aged care facilities |
| *10c) Principles of mobility goal setting, self-monitoring and feedback may support physical activity and sedentary behaviour change in the acute hospital setting.* | |
| n=25 (71.4%) | n=10  **Specificity of the recommendation:** suggest more specific; too prescriptive (other behaviour change techniques should be evaluated too)  **Scope for personalised modification:** depends on patient has the ability to self-monitor (e.g. dementia)  **Wording:** unclear meaning of statement; add action and coping  **Issues of implementation:** challenges of short hospital stays are too short to implement principles |
| ***Organisational factors*** | |
| *11) To address physical inactivity and sedentary behaviour during acute hospitalisation, work may be required to understand the complexity of underlying issues and approach hospital-system based solutions that consider the physical and social environment with other factors.* | |
| n=20 ^b^ (58.8%) | n=14  **Issues of implementation:** identification of barriers; ways for clinical staff to action; staffing levels  **Interaction with other recommendations:** subsequent statements help clarify, specifically link these  **Wording:** consider introduction of the term inactivity (define) or refer to ‘lack of movement’; revise phrasing “work may be required”, “underlying issues” and end of statement; unclear meaning of ‘hospital-based solutions’, ‘approach’ and ‘underlying issues’  **Example:** an example might help  **Structure of the recommendation:** suggest split into two recommendations |
| 12) T*o support physical activity and minimise sedentary behaviour in the acute hospital setting, consideration should be given to the potential value of policies; it may be relevant to address roles and responsibilities, logistics (such as transport and bed allocations) and adverse event reporting.* | |
| n=23 ^b^ (67.6%) | n=11  **Interaction with other recommendations:** redundant with *[recommendation 11]*  **Example:** an example would help; know who is responsible for what and role each of MDT; example of adverse event reporting rather than just/focus on falls  **Wording:** second part of statement is unclear (including ‘logistics’; context is unclear  **Issues of implementation:** reporting; consider organisational details; lack of planning/logistics for equipment associated with exercise  **Structure of the recommendation: p**ossibly split into two statements  **Coverage of concepts:** mention the people who need to be involved and their professional roles |
| *13) To support physical activity and minimise sedentary behaviour in the acute hospital setting, consideration should be given to the potential value of procedures; it may be relevant to address care plans and ward rounds, methods for prompting, and the use of devices for monitoring or assistance.* | |
| n=23 (65.7%) | n=12  **Example:** an example would help; detail of what devices  **Wording:** define “procedures”; unclear meaning of procedures (or protocols, clarity between the two required); unclear meaning “address”  **Interaction with other concepts:** may be possible to combine *[recommendations 11, 12, & 13]*; combine policies and procedures  **Issues of implementation:** may exist within a care bundle; whole of process, current models of care that maximise staff (e.g. medical) time rather than timing for patient needs  **Coverage of concepts:** good, including measures for encouraging activity, regularity of treatment, and consideration of digital technologies  **Scope for personalised modification:** needs to suit the individual patient. |
| 14) *Consideration should be given to the potential value of education as it relates to the shared responsibility of enabling physical activity and minimising sedentary behaviour in hospital (e.g. older adults, caregivers and relatives, developing and practicing health care professionals, people at different organisational levels).* | |
| n=27 (77.1%) | n=8  **Wording:** consider removing ‘potential’; make the statement less voluntary; education and training; clarify sort of education (who and in what); long (convoluted) statement  **Issues of implementation:** educational programs can be valuable and highly regarded; needs to lead to improved patient care |
| 15a) *Opportunities for physical activity and minimising sedentary behaviour should be incorporated into the daily care of older adults with a focus on function, independence and activities of daily living.* | |
| n=32 (91.4%) | n=3  **Example:** consider including examples, e.g., during personal hygiene, dressing  **Wording:** wording to complete the sentence (daily care of older adults)  **Issues of implementation:** implications for load/responsibility if only on physiotherapy |
| 15b) *Consideration should be given to moments for physical activity and minimising sedentary behaviour as part of common care events like mealtime, hygiene and dressing.* | |
| n=28 (80%) | n=7  **Interaction with other recommendations:** combine *[recommendation 15a and 15b]; [recommendation 15b]* is more explicit  **Wording:** replace ‘events’ with ‘activities’; fully incorporated into daily routine; unclear meaning of ‘moments’; add common social events like ward classes |
| 16a) *Consideration should be given to the influence of the built physical environment on the ability for older adults to be active.* | |
| n=21 (60%) | n=14  **Wording:** consider removing the word “built”; add indoors and out; modifications to suit patient requirements; active meaning physically, functionally and cognitively; required for safety; clarify meaning of “built physical environment”; vague statement  **Examples:** guidance on how to do/action |
| 16b) *Consideration should be given to any portable adaptations to the environment and equipment required for older adults to be active.* | |
| n=26 (74.3%) | n=9  **Interaction with other recommendations:** covered in previous statements.  **Wording:** add indoors and out; unclear meaning of statement and “portable adaptations”; not just ‘consideration’ (meaning action)  **Example:** provide examples  **Issues of implementation:** equipment including oxygen and transport |

^a^ endorsement determined as a positive participant response with wording such as “agree”, “strongly agree”, “no comment”, “support this statement”, “supportive”, “ok”, “good”, “very good”, “true”, “clear and understandable”. % endorsement was calculated with the number of respondents for each item as the denominator, thus being in n=35 for most items as no response was given by n=3 except where indicated.

^b^ no response by n=4 (denominator for % endorsement n=34)

^c^ no response by n=2 (denominator for % endorsement n=36)

**Figure S1:** Draft introductory material as presented to participants for feedback in Round 4

The following statements are proposed as recommendations (targets) that can be aimed for.

These recommendations were drafted based on an expert consensus process and should be considered as such within a hierarchy of evidence. Individually, experts may have used knowledge of research evidence to inform their responses throughout the process. However, the quality of evidence and strength of these recommendations has not been graded.

Currently there are no recommendations on how proposed targets should be measured or implemented. Any examples provided were generated from the consensus process and are for illustrative purposes only; further appraisal of examples and consideration of feasibility is suggested.

This expert consensus process has highlighted the value and importance of taking a person-centred approach to developing guidelines for physical activity and sedentary behaviour. This means that there may be circumstances when modification to targets are required. Circumstances for modification may include: medical instability or deterioration, contra-indications to activity or postures, physical factors (e.g., usual mobility, current ability and need for support), cognitive factors, goals of care (e.g., independence and optimised function, rest or sleep to specifically aid recovery, sensitive situations, end of life care), other safety concerns (e.g., falls) and barriers that relate to the environment (e.g., medical equipment, staffing).

**SUPPLEMENTARY MATERIAL REFERENCES**

1. Baldwin C, van Kessel G, Phillips A, Johnston K. Accelerometry shows inpatients with acute medical or surgical conditions spend little time upright and are highly sedentary: systematic review. *Phys Ther.* 2017;97(11):1044-1065.

2. Anderson JL, Green AJ, Yoward LS, Hall HK. Validity and reliability of accelerometry in identification of lying, sitting, standing or purposeful activity in adult hospital inpatients recovering from acute or critical illness: a systematic review. *Clin Rehabil.* 2018;32(2):233-242.

3. Lim SER, Ibrahim K, Sayer AA, Roberts HC. Assessment of physical activity of hospitalised older adults: a systematic review. *The journal of nutrition, health & aging.* 2018;22(3):377-386.

4. McCullagh R, Brady NM, Dillon C, Horgan NF, Timmons S. A review of the accuracy and utility of motion sensors to measure physical activity of frail, older hospitalized patients. *J Aging Phys Act.* 2016;24(3):465-475.
